# Cessation of Mass Drug Administration for Lymphatic Filariasis in Zanzibar in 2006: Was Transmission Interrupted?

**DOI:** 10.1371/journal.pntd.0003669

**Published:** 2015-03-27

**Authors:** Maria P. Rebollo, Khalfan A. Mohammed, Brent Thomas, Shaali Ame, Said Mohammed Ali, Jorge Cano, Alba Gonzalez Escalada, Moses J. Bockarie

**Affiliations:** 1 Centre for Neglected Tropical Diseases, Department of Parasitology, Liverpool School of Tropical Medicine, Pembroke Place, Liverpool, United Kingdom; 2 Ministry of Health, Zanzibar, United Republic of Tanzania; 3 Public Health Laboratory-IdC, Chake-chake, Pemba, Zanzibar, Tanzania; 4 London School of Hygiene and Tropical Medicine, London, United Kingdom; 5 Rey Juan Carlos University, Madrid, Spain; London School of Hygiene and Tropical Medicine, UNITED KINGDOM

## Abstract

**Background:**

Lymphatic filariasis (LF) is targeted for elimination through annual mass drug administration (MDA) for 4–6 years. In 2006, Zanzibar stopped MDA against LF after five rounds of MDA revealed no microfilaraemic individuals during surveys at selected sentinel sites. We asked the question if LF transmission was truly interrupted in 2006 when MDA was stopped.

**Methodology/Principal Findings:**

In line with ongoing efforts to shrink the LF map, we performed the WHO recommended transmission assessment surveys (TAS) in January 2012 to verify the absence of LF transmission on the main Zanzibar islands of Unguja and Pemba. Altogether, 3275 children were tested on both islands and 89 were found to be CFA positive; 70 in Pemba and 19 in Unguja. The distribution of schools with positive children was heterogeneous with pronounced spatial variation on both islands. Based on the calculated TAS cut-offs of 18 and 20 CFA positive children for Pemba and Unguja respectively, we demonstrated that transmission was still ongoing in Pemba where the cut-off was exceeded.

**Conclusions:**

Our findings indicated ongoing transmission of LF on Pemba in 2012. Moreover, we presented evidence from previous studies that LF transmission was also active on Unguja shortly after stopping MDA in 2006. Based on these observations the government of Zanzibar decided to resume MDA against LF on both islands in 2013.

## Author Summary

Lymphatic filariasis was highly endemic in Zanzibar when MDA commenced in 2001 to eliminate the disease. In 2006, Zanzibar, in the United Republic of Tanzania, was the first territory in Africa to complete five rounds of annual treatment using a combination of albendazole and ivermectin at 100% geographic coverage and achieving effective treatment coverage of over 65% during each round. MDA was stopped in 2006 after sentinel site surveys revealed parasite infection rates of zero in both humans and mosquito populations. In 2012, when new tools became available to verify the absence of transmission, we asked the question if transmission was truly interrupted when MDA was stopped in 2006. In January 2012, we performed the WHO recommended transmission assessment surveys (TAS) on the main islands of Unguja and Pemba to verify the absence of LF transmission in line with ongoing efforts to shrink the LF risk map. Altogether, 3275 children were tested on both islands and 89 were found to be CFA positive; 70 in Pemba and 19 in Unguja. The distribution of schools with positive children was heterogeneous with pronounced spatial variation on both islands. Based on the calculated TAS cut-offs of 18 and 20 CFA positive children for Pemba and Unguja respectively, we demonstrated that transmission was still ongoing in Pemba where the cut-off value was exceeded. We also presented evidence from previous entomological studies that LF transmission was active on Unguja shortly after stopping MDA in 2006. Based on these findings we concluded that LF transmission was still active in Zanzibar, and one million people at risk of acquiring LF, and recommended the resumption of MDA on both islands to eliminate the disease. In 2013, the government of Zanzibar decided to resume MDA with ivermectin plus albendazole on both islands.

## Introduction

Lymphatic filariasis (LF) is a major cause of acute and chronic morbidity and a significant impediment to socioeconomic development in 73 countries in Africa, Southeast Asia, the Americas, and the Pacific region. The World Health Organization (WHO) estimates that in 2012 more than 1.4 billion people living in these countries were at risk of acquiring the infection [1]. LF infection occurs through intense and long-term exposure to mosquito bites from several genera of anopheline and culicine mosquitoes that are carriers of the three parasites that cause human filariasis (*Wuchereria bancrofti*, *Brugia malayi* and *B*. *timori* [1]. The Global Programme to Eliminate Lymphatic Filariasis (GPELF) recommends annual mass drug administration (MDA) using albendazole in combination with either diethylcarbamazine (DEC) or ivermectin for 4–6 years as the main strategy to interrupt transmission of the disease [2]. From 2000 to 2012, more than 4.4 billion doses were used to treat over 500 million people in 56 countries, making the GPELF one of the most rapidly expanding global health programs in the history of public health [1]. Many countries implementing MDA have completed more than 5 consecutive annual treatment rounds but only China and South Korea have been verified by WHO for achieving elimination of LF [3]. In 2006, Zanzibar, in the United Republic of Tanzania, which started MDA in October 2001 [4], was the first country in Africa to complete five rounds of treatment using a combination of albendazole and ivermectin at 100% geographic coverage and achieving effective treatment coverage rate of over 65% during each round [3, 5]. A detailed description of the first round of MDA in 2001 and the subsequent four treatment rounds, including treatment coverage for each round, has been published previously [4, 5].

Parasite infection rates and intensities in the human and mosquito populations decrease after several rounds of MDA, but individuals may remain microfilaria and antigenemia-positive even after transmission has been interrupted [6]. A standard methodology called Transmission Assessment Survey (TAS) has been described by WHO to assess whether the prevalence of infection has been lowered to a level where recrudescence is unlikely to occur, even when MDA interventions have been stopped [6, 7]. After five rounds of MDA, an implementation unit (IU) is considered eligible for TAS if treatment coverage exceeds 65% on each round and the prevalence of microfilaraemia is below 1% on sentinel and spot check sites.

Treatment was stopped in Zanzibar in 2006, after five rounds of MDA and 2 sentinel and 12 spot-check site surveys in high risk urban and rural areas revealed parasite infection rates of zero in both humans and mosquitoes [3]. In line with ongoing efforts to shrink the LF map, we performed TAS in January 2012 to determine whether the successive rounds of MDA carried out between 2001 and 2006 achieved the interruption of disease transmission in the two main islands. Demonstrating the absence of active transmission of LF in Zanzibar is the first step in the verification process that could result in its reclassification as non-endemic and consequently, shrinking the LF map by yet another country.

## Materials and Methods

### Study area

Zanzibar is part of the United Republic of Tanzania located 35 km off the mainland Tanzania coast. It comprises two main islands, Pemba and Unguja, and a number of sparsely populated islets; the land areas of Unguja and Pemba are 1,654 km^2^ and 984 km^2^ respectively. About 1 million people live in Zanzibar with 65% of the inhabitants residing on Unguja. Unguja is the largest and most populated island of Zanzibar, with more than 40% of the population residing in Zanzibar town, the administrative and commercial centre of the two islands. Pemba, on the other hand, has three towns forming concentrated urban centres. Although Zanzibar is non-endemic for onchocerciasis, onchocerciasis transmission occurs in mainland Tanzania. Considering that there is free movement and settlement of the population in both the island and mainland communities, it was agreed to use ivermectin in combination with albendazole as the treatment regimen for LF [5]. In Zanzibar, the causative agent of LF is *Wuchereria bancrofti* which is transmitted by the urban mosquito, *Culex quinquefasciatus* [8, 9].

Prior to the first MDA round in 2001, parasitological and entomological surveys were carried out in two sentinel sites, Kizimkazi and Kwahani, in Unguja to collect baseline data for impact monitoring. The two sites represented the areas of highest risk of exposure to LF in rural and urban communities respectively in Zanzibar. Kizimkazi, which had a population of 3,037, is located in the dry arid stony area along the coast of the rural southern district. Pit latrines, cesspits and soakage pits, which serve as good breeding grounds for *Culex* mosquitoes, were common in Kizimkazi. Kwahani, located in the urban district of Unguja, had a population of 4,550 and featured many open drains where *Culex* mosquitoes breed. Before MDA started in 2001, the MF prevalence rates in Kizimkazi and Kwahani were 17.1% and 7.5% respectively, based on examining 500 people from each site [5]. The baseline surveys took place in September 2001 and were annually repeated before each MDA round. Finger prick blood samples (100μl) were obtained at night and examined using the counting chamber method as described in the WHO guidelines for MF surveys [7]. Treatment coverage rates over the 5 years varied from 76% (in 2001) and 83% during the subsequent 4 years with the last MDA round completed in November 2005. The prevalence of microfilaremia in the two sentinel sites dropped from 17.8% and 7.2% before treatment to 1.0% and 0.0% respectively after the fifth round [10].

### Transmission assessment surveys (2012)

Transmission assessment surveys (TAS) were performed in January 2012, six years after stopping MDA, using standard operating procedures (SOP) and WHO guidelines, which have been described in detail elsewhere [6, 7]. Zanzibar met the TAS eligibility requirements of having completed at least five effective rounds of MDA in all IUs with coverage >65% over the total population and MF rates <1% in each of the sentinel sites after five MDAs. It was decided to conduct the survey in two evaluation units (EU); one per island, as this would provide a clear picture of the current LF transmission status on each island while still having less than 2 million people on each EU. The TAS design was based on the net primary school enrolment rate, the target population size and number of schools on each island, and according to the rules established for *Culex-W*. *bancrofti* transmission areas [7]. A Microsoft Excel computer tool, the Survey Sample Builder (SSB) [7], was used to generate random number lists and inform TAS design calculations, including sample size and sampling intervals for the two TAS evaluation units. School surveys were conducted on both islands where net primary enrolment rates exceeded 75%. Children 6–7 years old were targeted for the TAS because antigenaemia in young children would reflect recent and active transmission, while antigenaemia in older children and adults may be related to infections that occurred before MDA. All children in grades 1 and 2 were eligible including a small proportion of those outside this age range and the oldest was eight years old. The sample sizes for the two evaluation units generated by the SSB were 30 school clusters on each island, which encompassed 1556 children in Pemba and 1684 children in Unguja.

The TAS critical cut-off value represents the threshold of infected individuals below which transmission is expected to be no longer sustainable, even in the absence of MDA.

If the total number of positive cases is at or below the critical cut-off value, the EU ‘passes’ the survey and MDA is not consider to be required [7]. TAS sample sizes and critical cut-off values are powered so that the EU has at least a 75% chance of passing if the true antigen prevalence is half the threshold level (2% for *Culex*, *Anopheles*, *and Mansonia* vector areas, and 1% for *Aedes* vector areas). In addition, there is no more than a 5% chance of passing if the true prevalence is greater than or equal to the threshold level. The critical cut-offs generated for Pemba and Unguja were 18 and 20 respectively.

### LF diagnosis

The Binax NOW Filariasis immunochromatographic card test (ICT) (Alere Inc., Scarborough, ME) was used to detect circulating filarial antigen (CFA) as described in the WHO guidelines [7]. Briefly, 100 μl of finger-prick blood was collected from each individual and then transferred to an ICT card test using a calibrated capillary tube. The test was read 10 minutes after closing the card, as instructed by manufacturers. A positive control filarial antigen was used to confirm the quality of the ICT cards. All positive controls turned out positive. The antigen contains the epitope present in circulating *Wuchereria bancrofti* antigen that is detected by the Binax Filariasis Now test. This was necessary to instil confidence in the large number of negative results expected. There was however a very minimal risk of false positives in Zanzibar which is not endemic for *Loa loa* [11]. ID number, class and test result of each child tested was recorded.

### Data entry and analysis

Data analysis was conducted using SPSS and the results were mapped using ArcGIS 10.1 (ESRI, Redlands, CA). The test results from each cluster in the different EUs were collected by the MOH survey team and the Public Health Laboratory Ivo de Carneri staff on Pemba. Data was entered using a Microsoft Access data base specifically developed by the Centre for Neglected Tropical Diseases (CNTD) in the Liverpool School of Tropical Medicine (LSTM) to support TAS. Two independent office workers entered data using a double entry system that automatically compare the two entries and detect any possible errors. Discrepancies were checked at CNTD for accuracy.

### Ethical approval and consent procedures

Ethical clearance for the study was granted by the LSTM Research Ethics Committee of the Liverpool school of Tropical medicine which approved the use of oral consent (Research Protocol 11.89RS). After approval from the Ministry of Education and school authorities, the MoH team met with the head master of each school to obtain permission to conduct the survey and to schedule a date for meeting with parents. The survey team explained the purpose of the surveys and received oral consent from teachers and parents. Written consent was not required for surveys conducted by the Ministry of Health as part of the disease control activities. Non consenting parents or non-assenting children could drop out from the study at that time or any time during the study.

## Results

**Fig 1:**
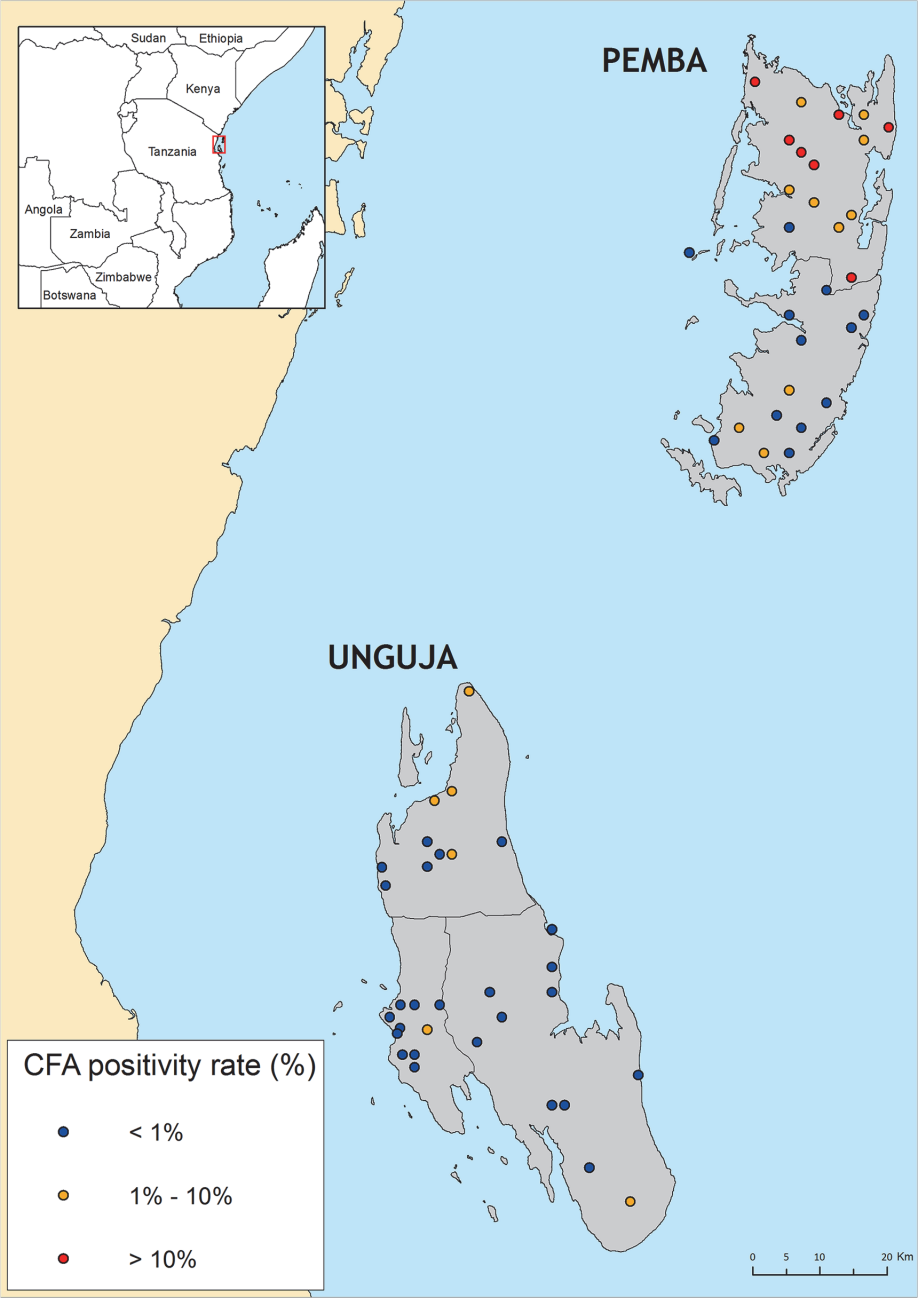
Spatial distribution and antigen positivity rates of schools selected for TAS surveys on Pemba and Unguja islands in Zanzibar, United Republic of Tanzania.

Altogether, 3 275 children were tested on both islands and 89 were found to be CFA positive. The CFA point prevalence for Pemba and Unguja were 5.4% (70/1298) and 0.9% (19/1977) respectively. Even though the sample size required for Pemba was 1556, we decided to discontinue the tests when the number of positive children largely exceeded the critical cut-off value of 18 for the EU. Some parents did not consent to including their children in the survey but no mop up was required since the critical cut-off was largely reached. The CFA prevalence for boys (0.91%) and girls (1.0%) was almost identical on Unguja, where the whole sample size was surveyed. The distribution of schools with antigen positive children was very heterogeneous on both islands with pronounced spatial variation between and within districts as shown in the Fig. 1.

A total of 30 schools, 9 from each of the four districts on Pemba Island were surveyed and CFA positive children were detected in every school with the exception of Cheke ckeke in south Pemba, where all 296 children were negative. Among the schools with positive children the CFA prevalence rates varied from 1.17% in Mkoani in South Pemba to 14.64% in Micheweni in North Pemba. The overall CFA prevalence for Pemba was 5.4% but the district specific CFA rates for Mkoani, Wete and Micheweni were 1.0%, 7.8% and 10.3% respectively.

In Unguja, where a total of 1684 children were tested, the total found to be CFA positive (19) was just below the critical cut off value of 20. The distribution of positive schools on the island was very heterogeneous with three (Central, North B and Urban) of the 6 districts revealing no positive children. Among the 3 districts with positive school, the district specific CFA rates were 4.7% (North A), 3.2% (South) and 0.4% (West).

## Discussion

Between 2001 and 2006, the Zanzibar programme for the elimination of LF carried out effective annual MDA campaigns to interrupt the transmission of the disease [5]. To effectively coordinate MDA in this predominantly Muslim country, the last Saturday of October was designated as the annual Filaria day (F-day) with a ‘mop-up day' on Sunday. Ivermectin in combination with albendazole were administered by highly motivated community drug distributors known as Filarial Prevention Assistants (FPAs) who ensured a high treatment coverage ranging from 70 to 80% for all five rounds. MDA was stopped in 2006 after sentinel site surveys revealed parasite infection rates of zero in both humans and mosquito populations as reported by Mohammed in his 2009 PhD thesis (Lymphatic Filariasis in Zanzibar: Epidemiology, Elimination and impact) with the University of Liverpool, United Kingdom [12]. The infection rates in humans and mosquitoes were determined by night blood survey and dissections respectively. It had been demonstrated in Egypt, where *W*. *bancrofti* was also transmitted by *Culex* mosquitoes, that five rounds of MDA using albendazole plus DEC can interrupt the transmission of LF in a population of 2.5 million [13].

Lymphatic filariasis was endemic on both islands before MDA commenced in 2001 as described in detailed investigations carried out many years prior the initiation of MDA [5, 9, 14]. Cross-sectional clinical, parasitological and entomological surveys for LF, conducted in urban and semi urban communities on Pemba in 1990 revealed that LF endemicity and vector species composition had not changed significantly for 15 years [9]. MF prevalence rates on Pemba during a survey conducted in 1975 ranged between 11.8% and 16.2% for people aged above 1 year [9]. Clinical manifestations in the form of hydroceole and lymphoedema were also common on the island, with prevalence of 22.4% and 1.4% respectively for adults above the age of 15 years [9]. Similarly, surveys conducted on Unguja in 1975 showed that the overall prevalence of clinical signs among men aged 15 years and older was 29.6% for hydrocele and 7.9% for elephantiasis, while the MF rates varied from 7.0% to 39.0% [12].

Our TAS results showed that five rounds of MDA in Zanzibar either failed to interrupt the transmission of LF on Pemba, where the TAS cut-off of 18 was surpassed by a huge margin early in the survey, or resurgence occurred after MDA was stopped in 2006. Unfortunately, the sentinel sites selected for monitoring the impact of MDA in Zanzibar did not include communities on Pemba and therefore the intensity of LF transmission on the island in 2006 could not be verified. Studies elsewhere have demonstrated that the vector of LF in Zanzibar, the highly efficient *Culex quinquefasciatus*, can sustain transmission in areas of low density microfilaraemia, even when MF is undetectable using traditional diagnostic methods based on around 60 μl [15]. In addition, recent studies on mainland Tanzania has also demonstrated that transmission of LF can persist after seven rounds of MDA in urban areas where *Culex quinquefasciatus* are the main vectors [16]. Infective mosquitoes were found in communities in India where MF rates dropped to zero after six rounds of treatment with DEC or ivermectin suggesting that transmission can occur in the absence of detectable MF if *Culex* mosquitoes are the vectors [15, 17].

Understanding the transmission dynamics of LF by different species of mosquitoes is essential for the rational planning of control measures and impact assessment. An important determinant of transmission efficiency is the genera of vector species involved [18]. For filariasis transmission to be interrupted, vector density or microfilaria intensity needs to be lowered below a threshold that ensures no new infections occur. This threshold for parasite density has been shown by quantitative models to be higher for anopheline which interact with *W*. *bancrofti* in a density dependent vector-parasite relationship known as facilitation. The relationships associated with culicine mosquitoes are known as limitation and proportionality and together with facilitation they describe the quantitative relation between microfilarial uptake and yield of infective L3 larvae in the mosquito vectors [18]. Based on these vector-parasite relationships, the TAS cut-off value for *Aedes* species (1%) is lower than that for *Anopheles* and *Culex* species (2%) [7]. The basis of grouping *Culex* with *Anopheles* species in determining these TAS cut-off values is unclear but analysis of eliminations threshold for *Anopheles* and *Culex* species suggest a higher threshold for the former [18]. The persistence of transmission in *Culex* transmission zones has led to growing concerns about the effectiveness of using MDA alone to eliminate LF without the inclusion of vector control [14, 19, 20]. On the other hand, once *Anopheles-*transmitted LF was eliminated in Solomon Island in the 1970s [20, 21], resurgence was never detected and it was declared non endemic by WHO in 2009 [22],

Vector control is effective against LF [20] but active vector control intervention did not resume in Zanzibar until after MDA for LF was stopped in 2006, when the Zanzibar Malaria Control Programme (ZMCP) started the distribution of free long lasting insecticidal nets (LLINs) targeting mainly pregnant mothers and children under the age of five years [23]. Bednet usage was initially lower in Pemba in comparison to Unguja but by 2008 every household in Zanzibar received two LLINs and, since 2006, six rounds of indoor residual spraying (IRS) have been conducted with synthetic pyrethroid lambda-cyhalothrin (ICON) resulting in over 90% coverage of all dwellings. IRS and LLINs target both endophilic (indoor resting) and endophagic (indoor feeding mosquitoes) mosquitoes including the vectors of LF on Zanzibar [20]. The combination of IRS and LLINs with other interventions resulted in a dramatic reduction of malaria prevalence in Zanzibar from 40% in 2005 to between 0.2 and 0.5% in 2011/2012 [23]. The low prevalence of LF infection in children in Unguja may be partly explained by the impact of the vector control measures as previous efforts to control LF in Unguja by vector control resulted in 65% reduction in mosquito density in houses [23]. The use of LLINs alone have resulted in the interruption of LF transmission in communities in Nigeria [24, 25] and Papua New Guinea [26]. Based on quantitative analysis of elimination thresholds for LF, the probability that the parasite will be eliminated following six rounds of MDA increases as the vector biting rates decrease [18].

The distribution of schools with antigen positive children was very heterogeneous on both islands with pronounced spatial variation between and within districts. Although Unguja barely passed the transmission interruption verification test by revealing fewer (19) CFA positive children than TAS cut-off of 20, four of the six positive schools had CFA positive rates higher than 5% and could enable transmission. Dissection of 6568 *Cx*. *quinquefasciatus* mosquitoes in 2006 found none to be infected with *W*. *bancrofti* but PCR assays on 5184 specimens collected between November 2007 and February 2008 showed a maximum likelihood infection rates of 1.13% (0.82% − 1.52%) that suggested ongoing transmission. It is therefore very likely that transmission was ongoing on the Island in 2006 when MDA was stopped [12].

The decision to stop MDA after several effective rounds of MDA should be based on statistically robust methodology. A recent multicenter evaluation to define endpoints for MDA in 11 countries concluded that TAS was superior to previous WHO guidelines used to determine when to stop MDA [6]. It was shown to be a practical and effective evaluation tool for stopping MDA although its validity for longer-term post-MDA surveillance will require the use of more sensitive tools for detection infection in humans and mosquitoes.

In conclusion, our findings in 2012 suggested that LF transmission was still active on Pemba. We also presented evidence from previous entomological studies that LF transmission was active on Unguja shortly after stopping MDA in 2006. Based on these findings including the heterogeneous distribution of CFA positive children in Unguja, and the high number of positives found compared (19) to the cut-off value (20) the government of Zanzibar decided to resume MDA with ivermectin plus albendazole on both islands in 2013. TAS will be repeated in 2015 after two rounds of treatment. However, the interpretation of the results from the TAS survey in 2015 may be confounded by the lack of treatment in Zanzibar for 7 years and pre-TAS sentinel site surveys in other evaluation units, including outside Zanzibar, may be required to determine if the criteria for TAS still holds.

## Supporting Information

S1 Checklist STROBE Checklist. (DOC)

S1 File Transmission assessment survey results for all participating schools in two evaluation units (EU). (PDF)
