# Supplementary material for: Cessation of Mass Drug Administration for Lymphatic Filariasis in Zanzibar in 2006: Was Transmission Interrupted?
Source: PLoS Negl Trop Dis. 2015 Mar 27;9(3):e0003669. doi: 10.1371/journal.pntd.0003669 (PMC4376862; doi:10.1371/journal.pntd.0003669)
Supplement: S1 File — (PDF) [file pntd.0003669.s002.pdf]

ICT CARD SURVEY ON LYMPHATIC FILARIASIS ON 36 SCHOOLS IN UNGUJA

The table shows number of school involved in the survey with their individual results

District	Name of the school	Total number of students screened			Total number of positive students			Total percentage
		Male	Female	Total	Male	Female	Total	
Central District	UROA	26	29	55	0	0	0	0
	DUNGA	27	28	55	0	0	0	0
	MARUMB I	27	28	55	0	0	0	0
	MWERA	26	29	55	0	0	0	0
	TUNGUU	27	28	55	0	0	0	0
	NDIJANI	22	33	55	0	0	0	0
North A District	NUNGWI	26	28	54	1	3	4	7.40%
	KIBENI	28	27	55	1	2	3	5.40%
	KIKOBWENI	28	27	55	2	0	2	3.60%
	PWANI MCHANGANI	28	27	55	0	0	0	0
	MKOKOTONI	27	28	55	2	2	4	7.20%
North B District	KINDUNI	28	28	56	0	0	0	0
	KIWENGWA	19	33	52	0	0	0	0
	BUMBWINI	28	27	55	0	0	0	0
	KIYONGWE	28	28	56	0	0	0	0
	MANGAPWANI	35	20	55	0	0	0	0
	DONGE	28	27	55	0	0	0	0
	DONGE KARANGE	27	28	55	0	0	0	0
South District	PETE	22	33	55	0	0	0	0
	KITOGANI	27	28	55	0	0	0	0
	PAJE	25	30	55	0	0	0	0
	MUYUNI	29	26	55	0	0	0	0
	KIZIMKAZI DIMBANI	34	21	55	0	0	0	0
	KUSINI	30	25	55	3	2	5	9.00%
Urban District	MWEMBEMAKUMBI	30	25	55	0	0	0	0
	JANGONOMBE	26	29	55	0	0	0	0
	RAHALEO	28	27	55	0	0	0	0
	MWEMBESHAURI	26	29	55	0	0	0	0
	MIGOMBANI	30	25	55	0	0	0	0
	CHUMBUNI	24	30	54	0	0	0	0
West District	REGEZA MWENDO	29	26	55	0	0	0	0
	LANGONI	26	29	55	0	0	0	0
	KIJITO UPELE	29	26	55	0	1	1	1.80%
	KISAUNI	28	27	55	0	0	0	0

	DIMANI	30	25	55	0	0	0	0
	MAGOGONI	29	26	55	0	0	0	0
	Total	987	990	1,977	9	10	19	0.90%

ICT CARD SURVEY ON LYMPHATIC FILARIASIS IN 36 SCHOOLS IN PEMBA

DISTRICT	SCHOOL	TOTAL	POSITIVES		Percentage
			M	F	
CHAKE CHAKE (South Pemba)	MADUNGU	22	0	0	**0.0%**
	MICHKAINI	19	0	0	**0.0%**
	VIKUNGUNI	52	0	0	**0.0%**
	VITONGOJI	16	0	0	**0.0%**
	FURAHA	37	0	0	**0.0%**
	MBUZINI	44	0	0	**0.0%**
	WESHA	20	0	0	**0.0%**
	MATALE	57	0	0	**0.0%**
	SHUNGI	29	0	0	**0.0%**
MKOANI (South Pemba)	MICHENZANI	51	0	0	**0.0%**
	MTUHALIWA	35	1	0	**2.9%**
	NG'OMBENI A	37	0	0	**0.0%**
	NG'OMBENI B	54	0	1	**1.9%**
	KIWANI	54	0	0	**0.0%**
	KANGANI	49	1	0	**2.0%**
	NGWACHANI	27	1	0	**3.7%**
	KENGEJA	41	0	0	**0.0%**
	MTAMBILE	49	0	0	**0.0%**
WETE (North Pemba)	JADIDA	50	0	0	**0.0%**
	KIZIMBANI	50	0	0	**0.0%**
	LIMBANI	34	0	2	**5.9%**
	MITIULAYA	24	1	3	**16.7%**
	FINYA	27	3	6	**33.3%**
	MZAMBARAUNI	45	1	1	**4.4%**
	CHWALE	18	0	1	**5.6%**
	OLE	35	6	2	**22.9%**
	MCHANGAMDOGO	50	1	2	**6.0%**
MICHEWENI (North Pemba)	MGOGONI	36	0	6	**16.7%**
	KINYASINI	39	3	6	**23.1%**
	MAKANGALE	25	3	0	**12.0%**
	WINGWI B	43	1	3	**9.3%**
	MICHEWENI	14	1	0	**7.1%**
	KIUYUMAZIWANG'OMBE	10	3	0	**30.0%**
	TUMBE	20	0	3	**15.0%**
	KONDE A	45	2	3	**11.1%**
	KONDE B	40	1	2	**7.5%**
	**TOTAL**	**1298**	**29**	**41**	**5.4%**
